# Selinexor, a selective inhibitor of nuclear export, enhances the anti-tumor activity of olaparib in triple negative breast cancer regardless of BRCA1 mutation status

**DOI:** 10.18632/oncotarget.28047

**Published:** 2021-08-31

**Authors:** Hélène Marijon, Sigal Gery, Hua Chang, Yosef Landesman, Sharon Shacham, Dhong Hyun Lee, Aimery de Gramont, Harold Phillip Koeffler

**Affiliations:** ^1^Cedars-Sinai Medical Center, Division of Hematology/Oncology, University of California, Los Angeles, CA 90048, USA; ^2^Department of Medical Oncology, Franco-British Hospital (Fondation Cognacq-Jay), Levallois-Perret, France; ^3^Karyopharm Therapeutics Inc., Newton, MA 02459, USA; ^4^Statistical Unit, Aide et Recherche en Cancérologie Digestive Foundation, Levallois-Perret, France; ^5^Cancer Science Institute of Singapore, National University of Singapore 117599, Singapore

**Keywords:** selinexor, XPO1, olaparib, triple negative breast cancer, BRCA1

## Abstract

Triple negative breast cancer (TNBC) is a deadly disease with limited treatment options. Selinexor is a selective inhibitor of nuclear export that binds covalently to exportin 1 thereby reactivating tumor suppressor proteins and downregulating expression of oncogenes and DNA damage repair (DDR) proteins. Olaparib is a poly (ADP-ribose) polymerase (PARP) inhibitor approved for the treatment of patients with breast cancer harboring *BRCA* mutations. We examined the effects of co-treatment with selinexor and olaparib in TNBC cell lines. *BRCA1* wildtype (*BRCA1*-wt) and *BRCA1* mutant (*BRCA1*-mut) TNBC cell lines were treated with selinexor and/or olaparib and effects on cell viability and cell cycle were evaluated. The effects of treatment were also evaluated in mouse xenograft models generated with *BRCA1*-wt and *BRCA1*-mut TNBC cell lines. Treatment with selinexor inhibited cell proliferation and survival of all TNBC cell lines tested *in vitro*. This effect was enhanced following treatment of the cells with the combination of selinexor and olaparib, which showed synergistic effects on tumor growth inhibition in MDA-MB-468-derived (*BRCA1*-wt) and MDA-MB-436-derived (*BRCA1*-mut) xenografts. As co-treatment with selinexor and olaparib exhibits anti-tumor activity regardless of *BRCA1* mutation status, the clinical implications of the combination warrant further investigation.

## INTRODUCTION

Breast cancer is one of the three most common cancers (together with lung and colorectal cancers) in the United States, accounting for 30% of female cancers [1]. Approximately 15–20% of breast cancers are triple-negative breast cancer (TNBC), which usually corresponds to basal breast cancer and is characterized by the absence of HER2, estrogen, and progesterone receptors. Patients with TNBC have poor prognosis with the worst disease-free and overall survival rates of all breast cancer types.

*BRCA1* and *BRCA2* are tumor suppressor genes responsible for the repair of double-strand DNA breaks (DSBs) via homologous recombination (HR) [2]. Germline *BRCA1* and *BRCA2* mutations have been identified in 10–20% of TNBC patients and 3–5% of TNBC patients harbor *BRCA* somatic mutations [3, 4].

Poly (ADP-ribose) polymerases (PARPs) are a group of enzymes activated by DNA damage. PARP1 and PARP2 assist in the repair of single-strand breaks (SSBs) through base excision repair. PARP inhibition traps the PARP-DNA complex at replication forks, leading SSBs to become DSBs that accumulate and ultimately lead to cell apoptosis if not corrected by appropriate repair mechanisms. As cells deficient in *BRCA1/2* cannot repair DSBs, they are particularly sensitive to the effects of PARP inhibition, resulting in synthetic lethality in tumor cells carrying the mutation while normal cells are spared [5]. Olaparib was the first PARP inhibitor (PARPi) approved by the United States Food and Drug Administration for the treatment of patients with deleterious germline *BRCA*-mutant, HER2-negative metastatic breast cancer who have been previously treated with chemotherapy [6]. Preclinical and clinical evidence have demonstrated that PARPi also affect cancer cells with other DNA repair mechanism defects (i.e., cells not harboring *BRCA1/2* mutations) [7]. Although 35% of all TNBC cases exhibit homologous recombination (HR) repair deficiency, in clinical practice, olaparib does not benefit TNBC patients without *BRCA1/2* mutations [8]. One strategy to expand the TNBC patient population that could benefit from this treatment is to combine PARPi with agents that induce DNA damage. PARPi have been shown to act as chemosensitizers and radiosensitizers but combination treatment of olaparib with chemotherapy (cisplatin, gemcitabine or irinotecan) has shown high toxicity [9–11]. PARPi can also be combined with less toxic targeted therapies that have overlapping effects in DNA damage repair (DDR) pathways.

The nuclear export protein exportin 1 (XPO1/CRM1) mediates the transport of over 200 proteins, including several key cell cycle regulators and tumor suppressors including APC, FOXO proteins, NPM1, p53, p21CIP, p27KIP1, BRCA1, and BCR–ABL. These proteins act as tumor suppressors when localized to the nucleus and enable cell proliferation and survival when exported to the cytoplasm [12–15]. XPO1 overexpression has been linked to poor prognosis and drug resistance in solid and hematological malignancies [16–18] including breast cancer [19].

Selinexor is an oral selective inhibitor of nuclear export (SINE) that binds covalently to cysteine 528 in the cargo binding pocket of XPO1 and inhibits its activity [20–22]. This inhibition causes accumulation of tumor suppressor proteins in the nucleus of malignant cells and blocks protein translation of oncogenes that drive cell proliferation, leading to cell cycle arrest and apoptosis of malignant cells [20–24].

Selinexor has demonstrated potent anti-cancer activities in multiple preclinical models of TNBC. The growth of 14 TNBC cell lines was inhibited by selinexor independently of PTEN, PIK3CA, TP53 or RAS mutation status. *In vivo*, selinexor reduced tumor growth by 42% (range 31 to 73%) in four patient-derived-TNBC xenograft models [25]. Furthermore, in an animal model of basal breast cancer, selinexor significantly reduced tumor cell growth to approximately one-third the volume of tumors observed in 5-fluorouracil-treated animals [14].

In clinical practice, selinexor as monotherapy was well tolerated in patients who received several lines of chemotherapy for TNBC and showed a clinical benefit rate of 30%, but no objective response [26]. As selinexor downregulates DDR proteins, it is mechanistically an ideal drug partner for PARPi, such as olaparib. The objective of the current study was to determine whether selinexor could increase the sensitivity of TNBC – with or without *BRCA1/2* mutations – to olaparib.

## RESULTS

### Synergistic anti-proliferative effect of selinexor with olaparib in a panel of TNBC cell lines

As different TNBC molecular subtypes often demonstrate different biological behavior, seven TNBC cell lines from representing subtypes were selected to evaluate the anticancer effects of selinexor and olaparib *in vitro* (Table 1). To examine the effects of selinexor and olaparib, various concentrations of olaparib – with or without selinexor – were applied to the cells for 72 hours and then cell viability was evaluated. Two *BRCA1*-mut cell lines, HCC-1937 and MDA-MB-436, showed very different sensitivity to olaparib, with MDA-MB-436 being the most sensitive cell line tested (IC50 17.5 μM) and HCC-1937 being the most resistant cell line tested (IC50 >300 μM).

**Table 1:** Characteristics of studied cell lines

Cell line	Subtype	BRCA1 status	TP53 status	Other mutations
**HCC1937**	TNBC BL1	MUT	MUT	MAPK13, MDC1
**MDA-MB-231**	TNBC MSL	WT	MUT	BRAF, CDKN2A, KRAS, NF2, PDGFRA
**MDA-MB-436**	TNBC MSL	MUT	MUT	
**MDA-MB-468**	TNBC BL1	WT	MUT	PTEN, RB1, SMAD4
**BT-549**	TNBC M	WT	MUT	PTEN, RB1
**Hs578T**	TNBC MSL	WT	MUT	HRAS, CDKN2A
**MDA-MB-453**	TNBC LAR	WT	WT	PTEN, CDH1
**BT474**	Luminal B (HER2+)	MUT	MUT	

Abbreviations: BL1: basal-like 1, LAR: luminal androgen receptor, M: mesenchymal, MSL: mesenchymal stem-like, MUT: mutated, TNBC: triple-negative breast cancer, WT: wildtype.

**Figure 1:**
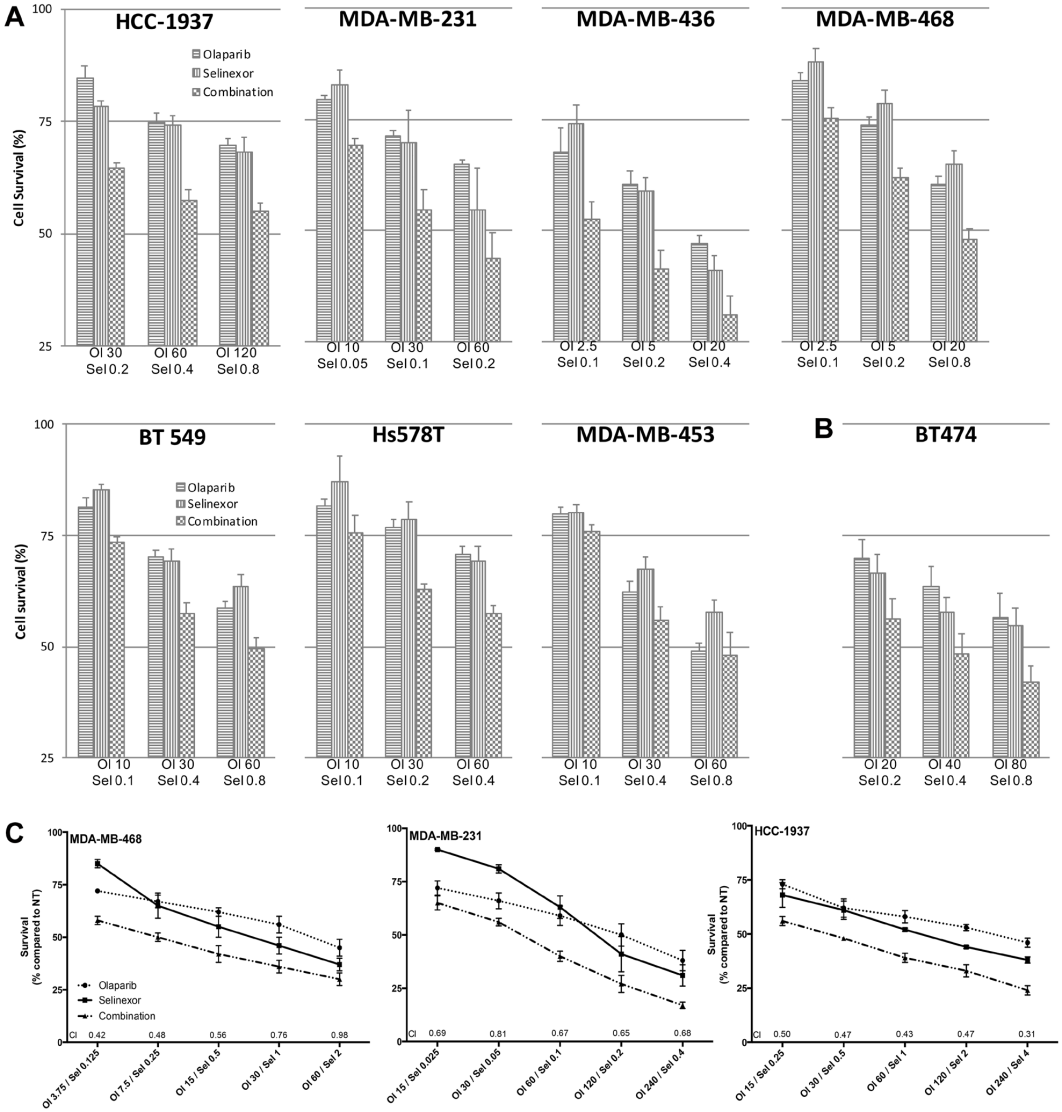
Synergistic anti-proliferation effects of selinexor and olaparib in a panel of TNBC cell lines. (**A**) Antiproliferation dose-response effect of olaparib and selinexor (alone and combined) in a panel of seven TNBC cell lines. Each bar reflects median ± standard error of the mean (SEM) of 3 experiments. Combination Index (CI) was determined using the CompuSyn software. (**B**) Antiproliferation dose-response effect of olaparib and selinexor (alone and combined) in a *HER2*-amplified BRCA-mutated breast cancer cell line (BT474). Each bar depicts median ± SEM of 3 experiments. (**C**) Cell survival after exposure to olaparib and selinexor (alone and combined) using a constant ratio between the drugs. Each data point indicates median ± SEM of 3 experiments. Concentrations in mM. Abbreviations: Ol: olaparib; Sel: selinexor; CI: combination index.

A non-constant drug ratio analysis (Figure 1A) showed synergism between selinexor and olaparib for all concentrations in all TNBC cell lines regardless of their *BRCA1* mutation status, except in MDA-MB-453 which showed additive effects. Interestingly, the ER-/HER2+ *BRCA1*-mut cell line, BT474 (Figure 1B), showed similar effects. Synergism between both drugs was confirmed by using a constant drug ratio analysis. For that analysis, MDA-MB-468, MDA-MB-231 and HCC-1937 were treated with 5 therapeutic combinations of olaparib – with or without selinexor—for 72 hours (Figure 1C). The combination index (CI) was lower than 1 in all 15 combinations. In the HCC-1937 and MDA-MB-468 cell lines, CI at ED50 was lower than 0.5 indicating high synergism.

### Selinexor enhances the effect of olaparib on apoptosis in *BRCA1*-wt and *BRCA1*-mut cell lines

**Figure 2:**
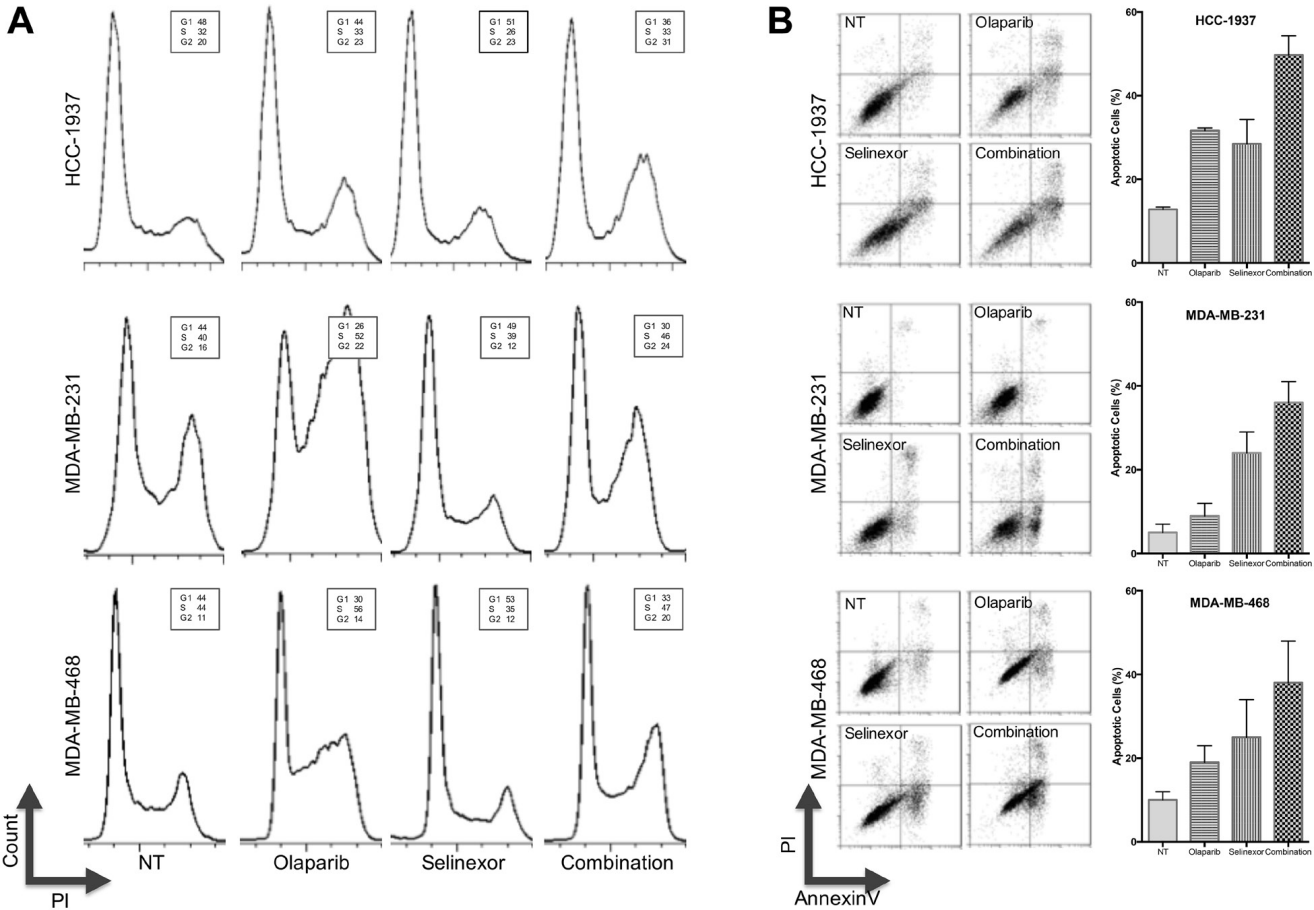
Effects of treatment with olaparib and selinexor (alone and in combination) on cell cycle and apoptosis of 2 BRCA1-wt TNBC cell lines (MDA-MB-231 and MDA-MB-468) and a BRCA1-mut cell line (HCC-1937). (**A**) Cell cycle analysis of HCC-1937, MDA-MB-231 and MDA-MB-468 cells. Cells were stained with PI and Annexin-V-FITC and analyzed by flow cytometry (representative results of 3 experiments). (**B**, left panel) Apoptosis of TNBC cells after exposure to olaparib and selinexor for 72 hours (alone and in combination). Cells were stained with PI and AnnexinV-FITC and analyzed by flow cytometry (representative results of 3 experiments). (B, right panel) For each cell line, the chart depicts the mean percentage of apoptotic cells ± SEM of 3 experiments.

Cell cycle and apoptosis analysis were performed following exposure of TNBC cell lines with *BRCA1*-wt (MDA-MB-231 and MDA-MB-468) and *BRCA1*-mut (HCC-1937) to olaparib and selinexor (alone or in combination). G2-arrest was induced as early as 24 hours after olaparib treatment of MDA-MB-231 and MDA-MB-468, and after 48 hours of HCC-1937 (Figure 2A). No significant effects on cell cycle were observed when the three TNBC cell lines were treated with selinexor alone. Interestingly, selinexor combined with olaparib increased the percentage of cells arrested at G2/M in the *BRCA1*-mut cell line HCC-1937 but not in *BRCA1-wt* cell lines MDA-MB-231 and MDA-MB-468 (Figure 2A).

Apoptosis studies showed that 9% of the MDA-MB-231 cells were apoptotic after exposure to olaparib for 72 hours, whereas 24% of the cells became apoptotic after exposure to selinexor. When the cells were exposed to both drugs, 36% of the cells became apoptotic after 72 hours. Similar results were seen for the other TNBC cell line expressing *BRCA1*-wt, MDA-MB-468, whereby 38% of cells underwent apoptosis after exposure to olaparib and selinexor for 72 hours, compared to 19% after treatment with olaparib and 25% following treatment with selinexor. Exposure of the *BRCA1*-mut HCC-1937 cell line to olaparib and selinexor for 72 hours resulted in apoptosis of 49% of the cells as compared to apoptosis of 32% of the cells after exposure to olaparib and 28% after exposure to selinexor for 72 hours (Figure 2B). Thus, co-treatment with olaparib and selinexor had an enhanced effect on apoptosis in all three TNBC cell lines, regardless of their *BRCA1* mutational status.

### Selinexor and olaparib synergistically inhibit the growth of *BRCA1*-mut MDA-MB-436 xenografts *in vivo*

Following the observed synergy of selinexor + olaparib *in vitro*, the effect of the combination was examined on MDA-MB-436 (*BRCA1*-mut) xenografts in immunodeficient mice. MDA-MB-436 cells were chosen due to the very slow growth of HCC-1937 *in vitro*. When tumor volumes reached 100 mm^3^, mice were randomly assigned to one of 4 different treatment groups: placebo, olaparib, selinexor or olaparib + selinexor. During treatment, digestive toxicities (diarrhea and weight loss >10%) were observed in mice receiving selinexor and selinexor + olaparib as early as day 5. Consequently, selinexor was stopped on Day 5 in both groups and reintroduced on Day 10 at a lower dose (5 mg/kg). Two mice were sacrificed prior to the end of the experiment: one in the selinexor + olaparib group due to weight loss >20%, and one in the olaparib group due to excessive tumor growth. One mouse in the control group died (the reason for death is unknown).

**Figure 3:**
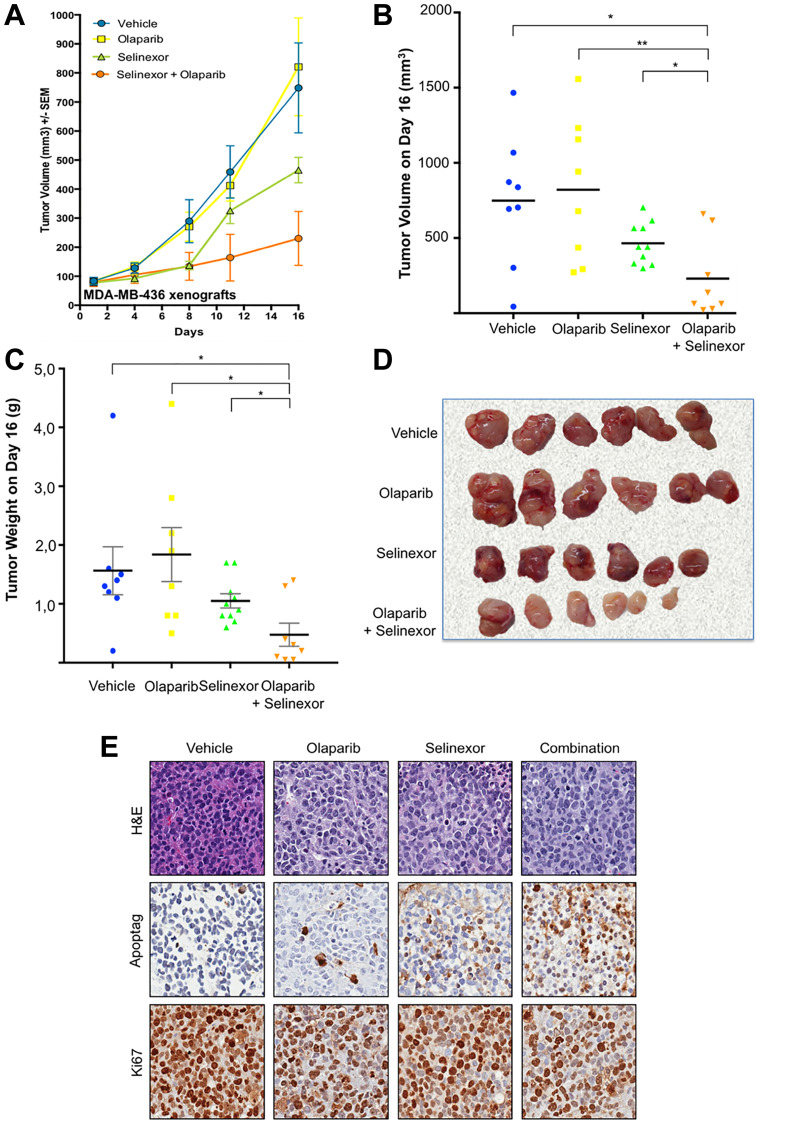
Synergistic effect of olaparib and selinexor on the growth of BRCA1-mut MDA-MB-436 xenografts in immunodeficient mice. (**A**) Volumetric growth of the tumor xenografts. Each data point represents mean tumor volume ± SEM. Drug doses and mode of administration: olaparib 50 mg/kg, intraperitoneal, 5 days/week; selinexor 7.5 mg/kg, oral, 3 days/week. (**B**) Tumor volume at the end of the experiment (Day 16). (**C**) Tumor weight at the end of the experiment (Day 16). (**D**) Representative tumors from each treatment group at the end of the experiment. (**E**) Representative images showing staining with H&E, ApopTag^®^ and Ki67 of tumors from each treatment group.

Tumor growth decreased in both the selinexor and combination group as early as day 4 whereas olaparib alone did not elicit this effect. From Day 10 onwards, selinexor + olaparib had a greater inhibitory effect on tumor growth compared to selinexor or olaparib alone (Figure 3A). At the end of the study, mean tumor volume was 748.5 ± 154.7 mm^3^ (8 tumors) with control, 821.0 ± 168.4 mm^3^ (8 tumors) with olaparib, 465.6 ± 44.2 mm^3^ (10 tumors) with selinexor, and 230.3 ± 92.9 mm^3^ (8 tumors) with selinexor + olaparib (*p* = 0.006 by ANOVA) (Figure 3B). Average tumor growth inhibition (TGI) compared to the control group was 42% with selinexor, and 79% with selinexor + olaparib (Figure 3A). Although no significant tumor growth inhibition was observed for the olaparib single agent treatment group when compared with the control, tumor volumes were significantly lower in the combination group compared to each of the single agent groups demonstrating synergistic effect of the combination (groups were compared 2 by 2 using a non-paired *t*-test).

Similarly, mean tumor weight was significantly lower with selinexor + olaparib (0.48 ± 0.2 g) compared to the other treatment groups (1.56 ± 0.41 g with control, 1.84 ± 0.46 g with olaparib, 1.05 ± 0.12 g with selinexor; *p* = 0.026 by ANOVA, Figure 3C).

Macroscopic analysis revealed that tumors receiving selinexor + olaparib were less vascularized than those receiving other treatments. Interestingly, the largest tumor in the selinexor + olaparib group was mainly necrotic (Figure 3D). This was confirmed by Ki67 staining in the 2 largest tumors from this group, which showed a thin layer of tumoral cells surrounding large areas of necrosis (data not shown).

Microscopic analysis using ApopTag^**®**^ staining confirmed an increase in apoptotic cells with selinexor compared to control, and with selinexor + olaparib compared to both monotherapies. Ki67 staining decreased with selinexor + olaparib compared to the 3 other treatments (Figure 3E).

### Selinexor and olaparib have synergistic effects *in vivo* on the growth of xenografts derived from MDA-MB-468, a *BRCA1*-wt TNBC cell line

**Figure 4:**
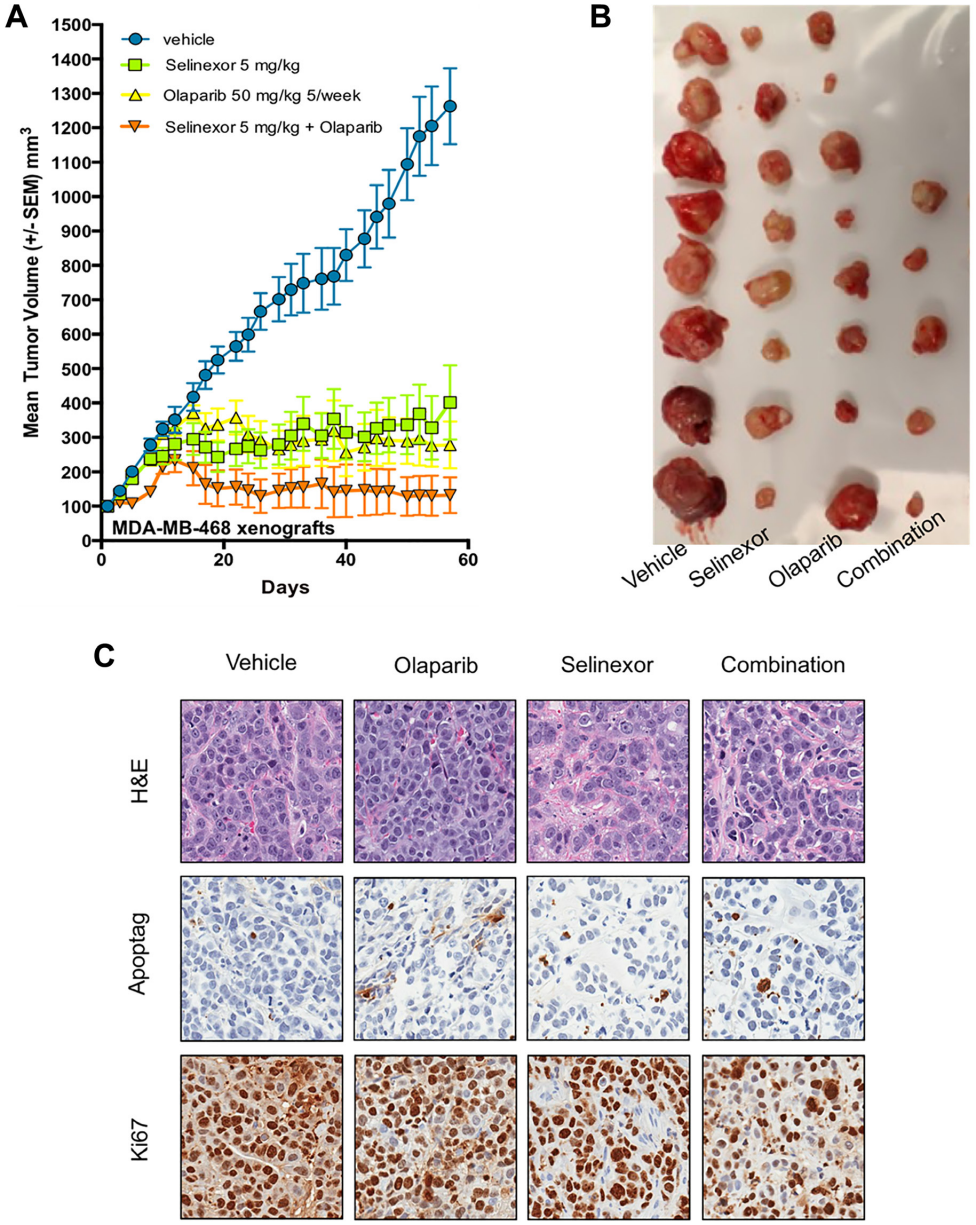
Synergistic effect of olaparib and selinexor on the growth of BRCA1-wt MDA-MB-468, xenografts in immunodeficient mice. (**A**) Volumetric growth of the tumor xenografts. Each data point represents mean tumor volume±SEM. Drug doses and mode of administration: olaparib 50 mg/kg, intra peritoneal, 5 days/week; selinexor 5 mg/kg, oral, 3 days/week. (**B**) Tumors from each treatment group at the end of the experiment. (**C**) Representative images showing staining with H&E, ApopTag^®^ and Ki67 of tumors from each treatment group.

We repeated the same experiment with xenografts derived from MDA-MB-468 (*BRCA1*-wt) except for the dose of selinexor (decreased to 5 mg/kg). As xenograft growth was slower, the experiment was stopped on Day 59. At the end of the experiment, mean tumor volume was 1346 ± 111 mm^3^ (SEM, 8 tumors) with control, 338 ± 108 mm^3^ (8 tumors) with olaparib, 384 ± 114 mm^3^ (8 tumors) with selinexor, and 136 ± 111 mm^3^ (5 tumors due to 3 complete responses) with selinexor + olaparib. Average TGI compared to control was 81% with olaparib, 77% with selinexor and 98% with selinexor + olaparib, indicating a synergistic effect of the combination treatment (Figure 4A). Treatment with selinexor + olaparib showed a statistically significant reduction in tumor growth relative to olaparib or selinexor alone (*p* = 0.0207 for both).

Interestingly, the effects of the combined treatment were observed as early as Day 20. Moreover, three complete responses were observed with selinexor + olaparib (Figure 4B).

Although a microscopic analysis did not reveal major changes after ApopTag^®^ staining, Ki67 staining was weaker with selinexor + olaparib compared to other treatments (Figure 4C).

### Effects of olaparib and selinexor on HR in *BRCA1*-wt TNBC cell line

**Figure 5:**
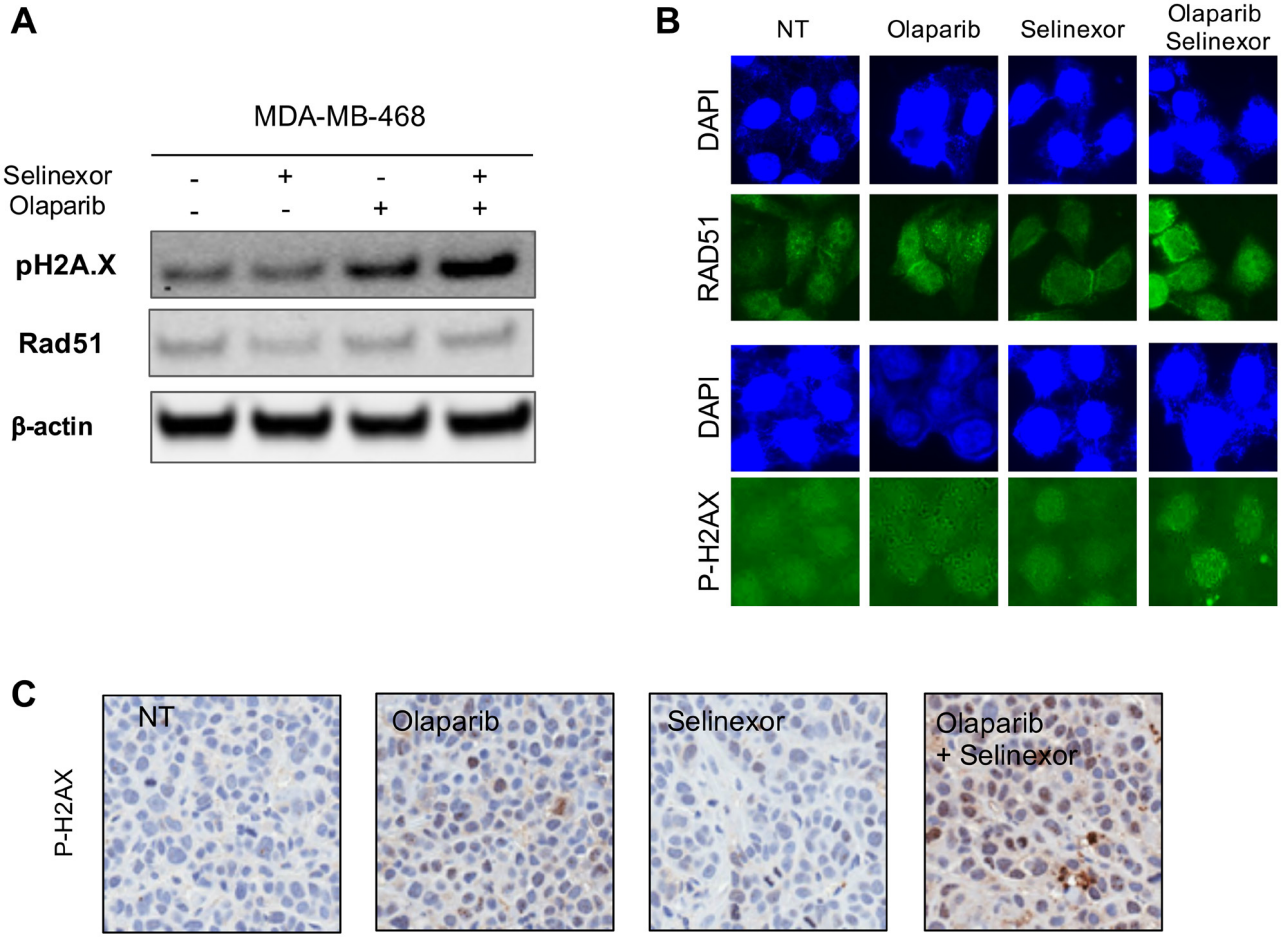
Effects of treatment with olaparib and selinexor (alone and in combination) on the homologous recombination pathway in MDA-MB-468 (BRCA1-wt TNBC cell). (**A**) Expression of RAD51 and P-H2AX in MDA-MB-468 cells. (**B**) RAD51 and P-H2AX protein expression by immunofluorescence in MDA-MB-468 cells exposed to olaparib and selinexor (alone or in combination) for 24 hours. (**C**) Immunohistochemistry of P-H2AX in representative tumors from each treatment group (xenografts were derived from MDA-MB-468 cells).

Additional experiments, focusing on the HR pathway in cells expressing wildtype BRCA1, were performed based on the mechanism of action of olaparib. RAD51, which plays a major role in HR of DNA during DSB repair and the H2A histone family member X that becomes phosphorylated on serine 139 in reaction to DNA DSB, P-H2AX, were analyzed by western blot and immunofluorescent staining. After treatment with selinexor, RAD51 expression decreased whereas no changes in P-H2AX expression were observed. After treatment with olaparib, P-H2AX expression increased. After exposure to both treatments, P-H2AX was further induced, suggesting lack of HR and DSB accumulation (Figure 5A). These observations were confirmed by immunofluorescent analysis of MDA-MB-468 cells (Figure 5B). A similar trend was observed following immunohistochemistry of MDA-MB-468-derived tumor xenografts: P-H2AX staining was significantly increased in tumors treated with selinexor + olaparib compared to the other groups (Figure 5C). These results suggest that the combination induced DNA damage in MDA-MB-468, which could partly explain the synergy observed between olaparib and selinexor.

## DISCUSSION

In search of effective treatment options for TNBC, we tested PARPi olaparib and SINE drug selinexor as single agents and in combination in seven TNBC cell lines comprising four different subtypes with or without *BRCA1* mutations. Synergistic effects of olaparib and selinexor on cell viability and proliferation were observed in all TNBC cell lines tested, regardless of their *BRCA1* mutation status or molecular subtypes. Synergy was also observed in HER2+ BT474 cells carrying a *BRCA1* mutation. Interestingly, although the TNBC cell line HCC-1937 carries a *BRCA1* mutation, it was the least sensitive cell line to olaparib among all cell lines tested, suggesting that factors other than *BRCA* mutations affect cancer cell sensitivity to olaparib, as also shown by other researchers [7]. The synergistic anti-tumor effects of olaparib and selinexor were also observed in mouse xenograft models of TNBC cell lines MDA-MB-468 (*BRCA1*-wt) and MDA-MB-436 (*BRCA1*-mut). Surprisingly, in the MDA-MB-436-derived xenograft model, we observed a trend for bigger tumors in the olaparib group. This difference was not statistically significantly different compared to the control group and appeared only on the last day of the experiment. Several reasons could explain this observation, the main one being the exponential tumor growth that led to stopping the experiment on day 16. Indeed, the effect of olaparib as monotherapy is generally observed only 10–15 days after the start of treatment, as was also seen in the *in vivo* experiment with MDA-MB-468-derived xenografts. Higher doses of olaparib in this TNBC model would likely induce tumor growth inhibition as a single agent. Subtherapeutic doses of single agent are often used in animal experiments designed to show synergy of combinations, and in this experiment, the synergy of selinexor and olaparib was obvious in the combination group despite a lack of efficacy with single agent olaparib at the dose tested.

DDR pathways form a complex, interacting defense mechanism against genotoxic damage. Deficient DDR is associated with increased mutational load and genome instability and often leads to neoplastic transformation and proliferation. Cells harboring DDR defects may rely on other repair pathways for survival, therefore inhibition of different DDR pathways can increase DNA damage, enhancing the sensitivity of cancer cells to therapy [27]. For this reason targeting DDR is an attractive therapeutic option [28].

Selinexor belongs to a unique class of drugs that selectively inhibit nuclear export; therefore, its mechanism of resistance may not be shared by other chemotherapeutic agents. This provides an opportunity to combine it with other therapeutic modalities such as PARPi to increase its effectiveness. The inherent complexity of the mechanism behind XPO1 inhibition involves the ability of XPO1 to interact with major tumor suppressors and cell cycle regulators, potentially targeting multiple pathways. Selinexor has been shown to downregulate multiple proteins involved in several DDR pathways, including HR [29], thereby functioning as a chemosensitizer and radiosensitizer. Cheng et al. showed that selinexor significantly reduced tumor cell growth to approximately one-third the volume of tumors observed in 5-fluorouracil-treated animals in an *in vivo* breast tumor model of basal breast cancer. Reduced tumor growth was associated with a reduced percentage of cellular proliferation and an increase in apoptosis markers [14]. Selinexor as monotreatment was well tolerated even in patients having received several lines of chemotherapy for TNBC and showed a clinical benefit rate of 30%, but no objective response [26]. Therefore, selinexor's more tolerable safety profile, compared with traditional chemotherapy, makes it a more desirable partner for combination therapy.

Kashyap et al. have shown that selinexor enhances the antitumor effects of DNA damage-inducing chemotherapies, in part through inhibition of DDR [29]. In addition to their role in DNA repair, PARPs are essential for many cellular processes, including chromatin remodeling, transcription, messenger RNA processing, and replication fork stabilization [30–32]. PARP inhibitors show synthetic lethality when combined with germline abnormalities in many DDR pathway components, such as mutations in ATM, ATR, BRCA1/2, CHK1, CHK2, PALB2, and RAD51 genes. Loss of several other genes involved in BRCA1-dependent HR repair, demonstrated in basal-like/TNBC cells also contribute to BRCA1-like features (sometimes termed BRCAness) [33] that confer sensitivity to olaparib. Tumors harboring DDR pathway aberrations that prevent HR repair of DSBs depend on PARP to repair SSBs and prevent them from progressing to irreparable, synthetically lethal DSBs [34].

The synergistic anticancer effects of olaparib and selinexor observed in TNBC cell lines regardless of their BRCA1 mutational status in our studies suggest that selinexor can potentially expand the patient population that could benefit from PARPi. In cancer cells without defects in DNA repair pathways, selinexor can reduce DNA repair proteins and sensitize cancer cells to PARP inhibition.

In addition to its effect on DNA repair pathways, several other mechanisms may contribute to the potent anti-tumor effects of selinexor in TNBC. Preclinical studies have shown that selinexor can inhibit proliferation and migration of TNBC cells by restoring the expression of arrestin-related domain-containing protein 3 (ARRDC3) [35]. Cheng et al. [14] showed that selinexor not only blocks the cytoplasm exportation of survivin—a multifunctional protein that can inhibit caspase-dependent apoptosis when it is localized in the cytoplasm—but also downregulates survivin transcription to promote apoptosis of TNBC cells.

Analysis of xenograft tumor growth derived from *BRCA1*-mutated and *BRCA1*-wildtype TNBC cell lines showed that co-treatment with selinexor and olaparib had a synergistic effect on tumor inhibition as compared to selinexor or olaparib alone. Tumors derived from *BRCA1* mutated cells showed increased apoptosis and decreased proliferation following combination treatment. Furthermore, tumors in mice co-treated with selinexor and olaparib were less vascularized than tumors in mice treated by a single drug, suggesting an effect of the combination treatment on inhibition of angiogenesis. Gravina et al showed significantly lower levels of angiogenetic cytokines interleukin 8 and vascular endothelial growth factor in prostate cancer cells treated with selinexor as compared to untreated controls [36].

Attiyeh et al. reported that selinexor induced a robust increase in p53 in Wilm’s tumor KT-10 xenografts with cleavage of PARP apparent after the first dose of drug [37].

RAD51 expression and the rate of RAD51-mediated HR are both elevated in many types of cancer, including breast cancer [38]. RAD51 facilitates metastatic dissemination in TNBC [39]; overexpression of RAD51 in *BRCA* mutated TNBC cell lines has been linked to cell survival and proliferation [40]. Silencing RAD51 in TNBC cell lines sensitize them to the PARP inhibitor BT-888 [41]. Furthermore, a defect in HR pathway was associated with synergism between olaparib and histone deacetylase inhibitors in TNBC [42]. Analysis of the expression of proteins involved in HR following treatment with selinexor and olaparib showed that selinexor decreased RAD51 expression in MDA-MB-468 cells, consistent with Kashyap et al. [29] who showed that selinexor reduced the expression of multiple DNA repair proteins, including RAD51, CHK1, MLH1 in multiple cell lines. P-H2AX expression increased significantly in these cells after exposure to selinexor + olaparib, confirming an increase in DNA damage [43].

In addition to TNBCs, co-treatment with selinexor and olaparib can potentially benefit patients with other cancer types. A phase Ib clinical trial investigating the safety of co-treatment with selinexor and olaparib in patients with advanced solid tumors is currently ongoing (NCT02419495).

In conclusion, combination of selinexor and olaparib induces robust anti-tumor activity *in vitro* and *in vivo* in TNBC cell lines with or without a *BRCA1* mutation. Our pre-clinical data support further investigation of the mechanism of action affecting this combination therapy in TNBC.

## MATERIALS AND METHODS

### Cell lines and culture conditions

Eight breast cancer cell lines (Table 1) were obtained from The American Type Culture Collection (ATCC). Each cell line was verified by short tandem repeat analysis when the study was initiated. Cells were tested for Mycoplasma annually by polymerase chain reaction. Cell lines were stored in liquid nitrogen and cultured for less than 6 months. The cells were cultured in Dulbecco’s modified Eagle’s medium/F-12 supplemented with 10% fetal bovine serum at 37°C and humidified 5% CO_2_. The cell line characteristics are shown in Table 1.

### Drugs and reagents

Selinexor was provided by Karyopharm Therapeutics Inc. Olaparib was obtained from LC Laboratories.

For *in vitro* experiments, selinexor and olaparib were solubilized in dimethyl sulfoxide (DMSO) and stored at −20°C until use. For *in vivo* experiments, selinexor was prepared in 0.6% w/v Pluronic F-68 surfactant and 0.6% w/v PVP K-29/32 polymer. Olaparib was supplied as a crystalline powder that was dissolved in DMSO equal to 10% of the final volume. Then Solutol HS-15 was added as 10% of the final volume, and a solution of 10% hydroxypropyl-β-cyclodextrin was added to bring the final volume to the desired quantity.

### Cell proliferation/viability assays

Seven TNBC cell lines (HCC-1937, MDA-MB-231, MDA-MB-436, MDA-MB-453, MDA-MB-468, Hs578T, BT-549) and one *HER2*-amplified *BRCA*-mut breast cancer cell line (BT474) were seeded in 96-well plates at densities of 3000–10000 cells per well depending on the growth characteristics of each cell line. After cells had adhered overnight, titrating concentrations of the designated drug (selinexor and/or olaparib) were added to the wells in triplicates and incubated at 37°C for 72 hours. Cell viability was then measured using an MTT (3-(4,5-dimethylthiazol-2-yl)-2,5-diphenyltetrazolium bromide) assay (Sigma-Aldrich).

Synergism of selinexor and olaparib was determined by combination index (CI) analysis adapted from the median-principle methods of Chou and Talalay [44]. CompuSyn 1.0 software was used for CI analysis (ComboSyn).

A CI lower than 1 defined synergism while a CI higher than 1 defined antagonism.

### Cell cycle analysis

Cell cycle analysis was performed in MDA-MB-231, MDA-MB-468 and HCC-1937 cells following exposure to selinexor and/or olaparib for 24–48 hours. MDA-MB-231 cells were treated with olaparib 60 μM and/or selinexor 0.2 μM for 24 hours. MDA-MB-468 cells were treated with olaparib 30 μM and/or selinexor 1 μM for 24 hours. HCC-1937 cells were treated with olaparib 60 μM and/or selinexor 1 μM for 48 hours. The cells were harvested and fixed with 70% ethanol at regular intervals. Fixed cells were stained with propidium iodide (PI) for flow cytometry analysis using BD FACScan (BD Biosciences, USA).

### Apoptosis assays

HCC-1937, MDA-MB-231 and MDA-MB-468 cells were plated, treated the following day with selinexor and/or olaparib in triplicates and incubated for 48 hours (MDA-MB-231 and MDA-MB-468) and 72 hours (HCC-1937). Following incubation, both floating and attached cells were collected and assayed for apoptosis using the Annexin V-FITC apoptosis detection kit (BD Biosciences) according to the manufacturer’s protocol.

### Analysis of *BRCA*-mut and *BRCA*-wt xenografts in immunodeficient mice

For the *in vivo* experiments, 5 × 10^6^ MDA-MB-436 (BRCA1 mutated) cells were suspended in 100 μL of phosphate-buffered saline (PBS) and mixed with 100 μL of Matrigel solution (Corning). The mixture was injected subcutaneously to the upper flanks of 5–6-week-old female nu/nu athymic nude mice (Harlan Laboratories). When tumors reached a volume of 100 mm^3^, the mice were randomly assigned to an experimental group (5 mice/group): (1) control group (placebo, i.e., vehicle without the drug); (2) olaparib group: the mice were injected intraperitoneally with olaparib 50 mg/kg 5 times/week; (3) selinexor group: the mice received oral selinexor 7.5 mg/kg 3 times/week; (4) selinexor + olaparib group: the mice received both olaparib and selinexor in the same dosage and mode of administration as in groups 2 and 3.

The tumors were measured twice weekly. Tumor volumes were determined using the formula A (length) × B^2^ (width) × 0.5236. The experiment was stopped on Day 16. At the end of the study, the mice were sacrificed, and the tumors were excised, weighed and fixed in 10% neutral buffered formalin and maintained in 70% ethanol. The fixed tumors were embedded in paraffin, sectioned and stained with hematoxylin and eosin (H&E) for histopathological examination.

A similar *in vivo* experiment was done with xenografts derived from MDA-MB-468 (*BRCA1*-wt) cells. The experimental design was the same, except for selinexor dosage which was 5 mg/kg, 3 times/week. The experiment was stopped on Day 59.

Animal experiments with MDA-MB-436-derived xenografts were performed at Cedars-Sinai Medical Center and strictly followed the guidelines of Cedars-Sinai Medical Center and the National Institutes of Health. Animal experiments with MDA-MB-468-derived xenografts were performed by Pharma Models, LLC., and all protocols and procedures were approved by the Institutional Animal Care and Use Committee.

### Analysis of protein expression in MDA-MB-468 cells

MDA-MB-468 cells were exposed to either drug-free medium or olaparib and/or selinexor. After different durations of exposure, cells were lysed with RIPA buffer (Millipore Upstate, USA) supplemented with protease inhibitor cocktail. For analysis of phosphorylated protein, a phosphatase inhibitor cocktail (PhosSTOP, Roche, Switzerland) was added to the lysis buffer. 40 μg proteins were separated by SDS-PAGE, transferred to nitrocellulose membrane, and blotted with primary antibodies p-H2AX (Cell Signaling Technology, USA) and RAD51 (Santa Cruz Biotechnology, USA); β-actin was used as a loading control.

### Immunofluorescence

Protein expression of RAD51 and p-H2AX were also evaluated by immunofluorescence in MDA-MB-231 and MDA-MB-468 cells after exposure to olaparib and selinexor (alone or in combination) for 24 hours: MDA-MB-231 cells were exposed to 0.2 μM selinexor and/or 60 μM olaparib. MDA-MB-468 cells were exposed to 1 μM selinexor and/or 30 μM olaparib. Sterile cover slips were placed in 12 or 24-well plates, washed with PBS and then with culture media. The coverslips were seeded with cells at a density of 10,000 cells/cm^2^ and incubated for 16 hours. The next day the cells were rinsed with complete PBS, fixed for 30 minutes with 4% formaldehyde in PBS, and then washed gently twice with PBS. The cells were quenched with 50 mM NH_4_Cl for 15 minutes, then washed with PBS for 4 minutes and blocked for 1 hour at room temperature with 1% bovine serum albumin. The cells were then incubated with primary antibodies p-H2AX (Cell Signaling Technology, USA) or RAD51 (Santa Cruz Biotechnology, USA) for one hour at room temperature or overnight at 4°C. After incubation with the primary antibody, the cells were washed 3 times with PBS and incubated with secondary antibody (Cell Signaling Technology, USA) for 1 hour. After incubation with the secondary antibody, the coverslips were washed 3 times with PBS. Cell nuclei were counterstained with DAPI (diamidino-2-phenylindole). An antifading agent was added (Fluoromount/Slow Fade/Vectashield) and the coverslips were mounted on clean glass slides and sealed.

### Histology and immunohistochemistry analysis

Xenograft samples from mouse models were fixed in 10% neutral buffered formalin and paraffin embedded. Sections cut at 5 μm were stained by routine H&E for histology analysis. Immunohistochemistry (IHC) staining was performed as previously described [45]. Apoptosis was detected with the ApopTag kit (Millipore, Cat No. S710003). The Ki67 antibody (Cell Marque, Cat No. 275R-18) and the p-H2A.X antibody (Millipore, Cat No. 05–636) were used for IHC. Digital images of the slides were obtained through an Aperio AT Turbo scanner at 20×.

## Abbreviations

*BRCA1*-mut: *BRCA1* mutant
*BRCA1*-wt: *BRCA1* wildtype
CI: combination index
DDR: DNA damage repair
DMSO: dimethyl sulfoxide
DSB: double-strand DNA break
HR: homologous recombination
H&E: hematoxylin and eosin
PARP: poly (ADP-ribose) polymerase
PARPi: poly (ADP-ribose) polymerase inhibitor
PBS: phosphate buffered saline
SEM: standard error of the mean
SSB: single strand DNA break
TGI: tumor growth inhibition
TNBC: triple-negative breast cancer
